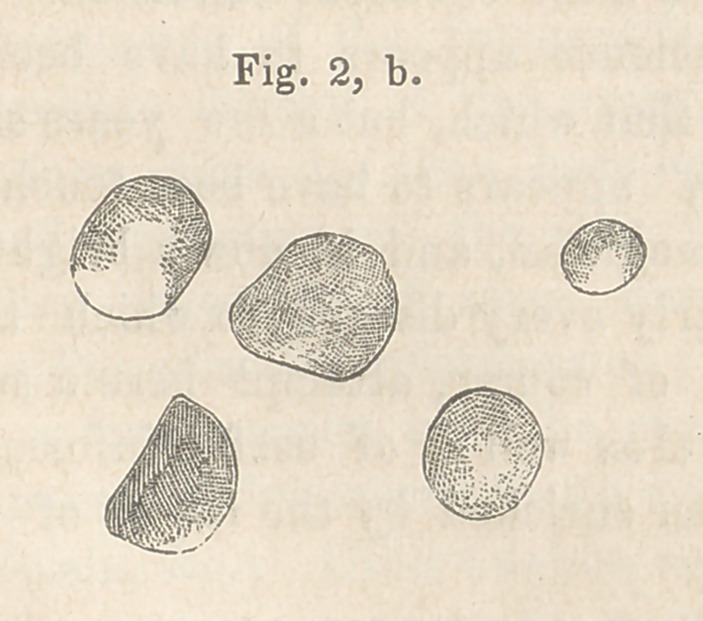# A Case of Curious Cutaneous Disease

**Published:** 1852-02

**Authors:** S. Weir Mitchell

**Affiliations:** Philadelphia


*A Case of Curious Cutaneous Disease.* By S. WEIR MITCHELL, M. D., of Philadelphia.

Jane ——, æt. 13. She had enjoyed good health up to a period about one year previous to the present date, when an eruption of a very peculiar character appeared upon the fore part of her neck, chin, and lips, and, somewhat later, upon the back of her neck. The eruption is but slight, twenty or thirty vesicles being constantly present, and running their course, to be succeeded by others of like nature. Their long continued presence seemed to have exerted no influence upon the little patient's general health; and save the local disfigurement and the constant itching which they occasion, the case might be passed by as one rather to be cured by time than attacked by remedies.

On the 20th of November, the child was brought by the mother to the clinic of the Jefferson Medical College, where I noticed the case in an ante-room. On this, and on several other occasions, I studied the disease with some care, and twice examined the contents of the vesicles with the aid of a microscope. When first seen, the eruption presented the following appearance: Around the lip were about a dozen of minute white vesicles, with semi-transparent apices filled with a thin serous fluid. These were also to be seen at a more advanced stage, when they began to assume an umbilicoid appearance, like the pustules of small pox, save that they still retained their white hue. In most of the papules, the central prominence now began to harden and to grow outwards; sometimes offering an irregular elevation, at others presenting the singular phenomenon of a pretty regular projection, shaped precisely like a minute finger, and growing from beneath the central elevation, or a little to one side of it. Some of these small horn-like growths stood directly out from the skin, while others hung downwards, being bent at their base. Their length varied greatly, the largest being about two and a half or three lines long.

After a few days, these prominences dried up and fell off, while the base gradually disappeared, the whole process from beginning to end occupying from a week to ten days.

I secured specimens of the eruption by seizing these projections with forceps and pulling them off. They seemed to draw out of the skin beneath which they appeared to grow, pushing before them, as a cover, the thin membrane of the vesicle. A careful section presented the appearance which I have drawn:

Fig. 1, a.

The fluid contents of the papule were a thin serum, in which the microscope discovered floating a number of yellowish globules, perfectly homogeneous and without nuclei. The peculiar prominences spoken of above, were formed of a mass of these globules, altered by drying, and changed in form by their close juxtaposition.

Fig. 2, b.

These albuminoid bodies varied so greatly in size as to render any measurements useless. They seemed to spring from the skin, and resembled greatly the globules of concrete albumen, which are met with at times in tumors, and not unfrequently upon the skin, in the neighborhood of pimples which are drying up, etc.

Under the use of arsenic and an ointment of equal parts of mercurial, tar, and sulphur ointments, the eruption rapidly changed its nature to that of a well developed squamous affection which grew better, and indeed, seemed in process of cure when I last saw it.

Another case of a similar nature fell under the notice of my friend, Mr. Brinton, about one year ago ; but, with this exception, I know of none other, and I have searched for it in vain among the English and French delineations and descriptions of cutaneous disease.

## Figures and Tables

**Fig. 1, a. f1:**
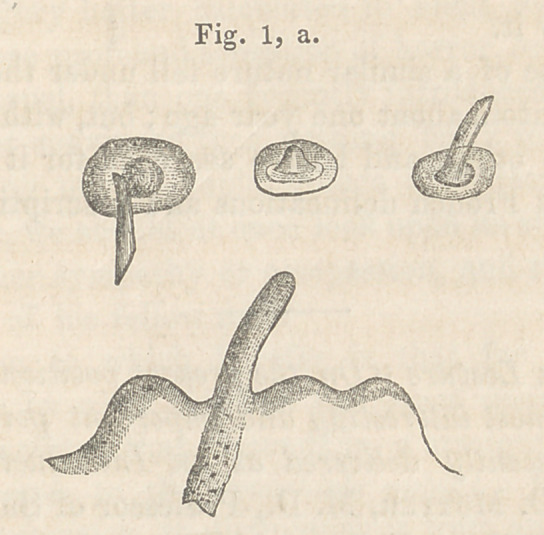


**Fig. 2, b. f2:**